# Knowledge, attitudes and screening practices regarding prostatic diseases among men older than 40 years: a population-based study in Southwest Nigeria

**DOI:** 10.11604/pamj.2017.27.151.10605

**Published:** 2017-06-30

**Authors:** Rufus Wale Ojewola, Ezekiel Sofela Oridota, Olanrewaju Samuel Balogun, Ezra Olatunde Ogundare, Taiwo Opeyemi Alabi, Oluseyi Omotola Banjo, Adeyinka Laoye, Babatunde Adetunmbi, Bamidele Oludele Adebayo, Rotimi Oluyombo

**Affiliations:** 1 Department of Surgery, College of Medicine of University of Lagos/Lagos University Teaching Hospital, PMB 12003, Idi-Araba, Surulere, Lagos, Nigeria; 2 Department of Community Health and Primary Care, College of Medicine of University of Lagos/Lagos University Teaching Hospital, PMB 12003, Idi-Araba, Surulere, Lagos, Nigeria; 3 Department of Paediatrics, Ekiti State University Teaching Hospital/Ekiti State University, Ado-Ekiti, Ekiti State, Nigeria; 4 Department of Surgery, Federal Teaching Hospital, Ido-Ekiti, Ekiti State, Nigeria; 5 Department of Medicine, Federal Teaching Hospital, Ido-Ekiti, Ekiti State, Nigeria

**Keywords:** Awareness, knowledge, attitudes, screening practices, prostatic diseases, Nigerian men

## Abstract

**Introduction:**

Despite the global increase in awareness of prostatic diseases resulting from widespread availability of screening tools, there is no evidence that the knowledge, attitudes and screening practices of Nigerian men have improved regarding prostatic diseases.

**Methods:**

A descriptive cross-sectional study amongst 305 community-dwelling men. Respondents were selected using multi-staged sampling techniques. Knowledge, attitudes and screening practices were determined based on responses to a semi-structured KAP questionnaire. Data were analyzed using SPSS version 18. Pearson's chi-square and Fisher's exact test (two-tail) with level of significance set at 0.05 were used to determine the level of statistical significance. Pearson's correlation coefficient was used to establish correlation between variables.

**Results:**

Mean age of respondents was 63.4±11.8 years. Slightly less than half, 145(47.5%) were aware of prostate cancer (PCa) while only 99(32.5%) and 91(29.8%) were aware of BPH and prostatitis respectively. About a quarter (25.1%) had heard of PSA. The main sources of information were radio and television. Overall, 143(46.9%) respondents had good knowledge while 162(53.1%) had poor knowledge. Sexually transmitted disease was the commonest misconception as the cause of prostatic diseases. Overall, 44.3% had good attitudes. Only 31(10.2%) respondents had ever carried out screening for PCa. Only educational and occupational status had significant associations with level of knowledge and attitudes of participants. The only factor that influenced screening practices was educational status.

**Conclusion:**

There is a poor level of knowledge, attitudes and screening practices regarding prostatic diseases in Nigeria. We recommend a widespread public health education to improve knowledge, attitudes and screening practices for prostatic diseases.

## Introduction

With the “graying” of the “baby boomers”, awareness of prostatic diseases has increased dramatically [1]. In particular, prostate cancer (PCa), which is now the most common male malignancy and other prostatic diseases such as benign prostatic hyperplasia (BPH) and prostatitis, are widely discussed in the media and are the subject of increasing interest from family physicians and the general public alike. Prostate cancer is a significant healthcare problem due to its high incidence and mortality and the cost associated with its detection and treatment [2, 3]. The awareness of prostatic diseases has increased in recent times with the recognition of early detection of PCa as a key factor in reducing mortality and morbidity. It is however sad that majority of patients in our sub-region present with locally advanced and metastatic diseases [4–6]. Several reasons have been suggested for this trend including a low level of awareness and knowledge, erroneous beliefs about prostatic diseases and poor healthcare-seeking behaviour of patients amongst other factors related to available facilities and poverty. Even though prostatic diseases have received much media attention, studies of the public's knowledge, perceptions or screening practices are not many in Nigeria especially in the rural communities where more than half of Nigerians live [7]. Recently, there has been growing interest in the role of knowledge, attitudes and screening practices in PCa prevention and control [8, 9]. A good knowledge or understanding of diseases is generally associated with a better healthcare-seeking attitude and behaviour [10]. Therefore, efforts at improving awareness about diseases should not be directed at malignant diseases only as efforts towards benign conditions which are by far commoner than malignant ones will also reduce morbidity and mortality significantly. Despite the global increase in awareness as a result of widespread availability of screening tools for prostate diseases, there is no evidence that the knowledge, attitudes and screening practices of Nigerian men have changed regarding prostatic diseases. The aim of the study therefore, is to determine the level of awareness, knowledge and attitudes towards treatment for prostatic diseases as well as screening practices amongst community-dwelling men.

## Methods

***Study design, setting and population***: This was a questionnaire-based descriptive cross-sectional study carried out amongst 305 male adults in the Ido/Osi Local Government Area (LGA) of Ekiti State, Southwest, Nigeria. This LGA is basically an agrarian community with thirteen major communities. The target population was male adults above 40 years of age.

***Ethical issues***: Approval for the study was obtained from the Health Research and Ethics Committee of Lagos University Teaching Hospital, Lagos. Further approval was obtained from the Executive Chairman of the LGA as well as the traditional rulers of the communities used for the study. Consent was also sought and obtained from each participant.

***Sample size determination***: The sample size for the study was calculated using the formula: n= z^2^pq/d^2^. Where: n = desired sample size when population > 10,000, z = level of significance at 95% CI (=1.96), p = proportion of the study population who are aware of prostate cancer and screening from similar previous study = 0.22 [11], q = 1–p = 0.78 and d = degree of accuracy desired, usually set at 0.05. Sample size (n) = z^2^pq/d^2^ = (1.96)^2^ x (0.22) x (0.78)/ (0.05)^2^ = 3.84 x 0.22 x 0.78/0.0025 =0.6589/0.0025= 264. The minimum sample size required for this study was 264.

***Sampling techniques***: Participants were recruited using a multi-stage sampling technique. This involved the following stages: firstly, the selection of 3 of the 11 electoral wards in the study area. This was followed by the selection of 1 community from each selected ward. Thirdly, 15 to 20 streets were chosen in each community. These first 3 stages were carried out using a simple random sampling technique by balloting. Fourthly, about 5 to 15 houses in these streets were selected by systematic random sampling depending on the length of the street. Finally, an individual was chosen for the survey by simple random technique amongst all men above 40 years of age in each selected house. All men selected who consented to participate were recruited for the study.

***Data Collection***: The date of the data collection was announced a week before data collection in the three selected communities using public address system. Each participant was interviewed in their house by the surgical resident doctors of Federal Teaching Hospital, Ido-Ekiti about their awareness and knowledge of prostatic diseases as well as their attitudes and behaviour towards treatment and screening for prostatic diseases. A semi-structured and pre-tested knowledge, attitudes and practices (KAP) questionnaire containing sections on demography, knowledge, attitudes and screening practices regarding prostate diseases was used as the instrument for data collection.

***Data analysis***: Data from the 305 men were analyzed with SPSS version 18 using simple proportion and the results were displayed in tables and chart. Knowledge about prostatic diseases was determined based on responses to 26 knowledge-based questions. Respondents with 0-13 correct responses were adjudged to have poor knowledge while those with 14-26 were adjudged to have good knowledge. Attitude of participants to prostatic diseases were judged based on the appropriateness of their responses to the six questions on attitude to early presentation and treatment of prostatic diseases. Each was scored 1-5 with 1 being the least appropriate and 5 being the most appropriate. Scores of 5.0-17.4 were adjudged to be negative attitude while scores of between 17.5 and 30.0 was adjudged to be positive. Screening practices were also determined. Relationships between variables were determined using Pearson's chi-square and Fisher's exact test (two-tail) with level of significance set at 0.05 (p < 0.05) to determine level of statistical significance. Pearson's product-moment correlation coefficient was used to establish correlation between variables.

## Results

A total of 319 adult males older than 40 years were recruited but only 305 men completed the questionnaires and had adequate data for analysis. This translates to a response rate of 95.6%.

***Demographic profiles***: Ages of the participants ranged from 41-90 years with a mean age of 63.4 ± 11.8 years. The majority, 269 (88.2%) of the participants were married while the remaining 36 (11.8%) were either single, divorced or widowers. The majority were of Yoruba tribe (95.7%) and of Christian religion (84.3%). Two hundred and forty-one (79%) participants had at least primary education while 64 (21%) had no formal education. Ninety-five (31.1%) were farmers, 74 (24.3%) were civil servants and 61 (20.0%) were artisans. The remaining 75 (24.6%) included pensioners, business men and men of other occupations.

***Awareness of prostatic diseases and screening***: Slightly more than half, 158 (51.8%) respondents had heard of prostatic diseases before the time of this study. Slightly less than half, 145 (47.5%) were aware of PCa while less than one-thirds, 99 (32.5%) and 91 (29.8%) were aware of BPH and prostatitis respectively. Only a quarter (25.1%) had heard of prostate specific antigen (PSA) as a screening tool for PCa. The main sources of information amongst those who were aware of prostatic diseases were radio and television in 59.5 and 49.4% respectively. Other details about awareness and sources of information are depicted in Table 1.

**Table 1:** Awareness and sources of information about prostatic diseases

Variable	Frequency (n)	Per cent (%)
**Awareness about prostatic diseases**		
Yes	158	51.8
No	91	29.8
Don’t know	56	18.4
**Awareness of BPH**		
Yes	99	32.5
No	147	48.2
Don’t know	59	19.3
**Awareness of prostate cancer**		
Yes	145	47.5
No	131	43.0
Don’t know	29	9.5
**Awareness of prostatitis**		
Yes	91	29.8
No	164	53.8
Don’t know	50	16.4
**Awareness of screening for prostate cancer**		
Yes	96	31.5
No	115	37.7
Don’t know	94	30.8
**Awareness of PSA screening**		
Yes	77	25.2
No	121	39.7
Don’t know	107	35.1
*Source of information among 158 respondents		
Friends	51	32.3
Family	46	29.1
Radio	94	59.5
Television	78	49.4
Newspaper	59	37.3
Magazines	18	11.4
Hospital	47	29.7
Church	44	27.8
Others	27	17.1

* Multiple responses possible

**Figure 1:**
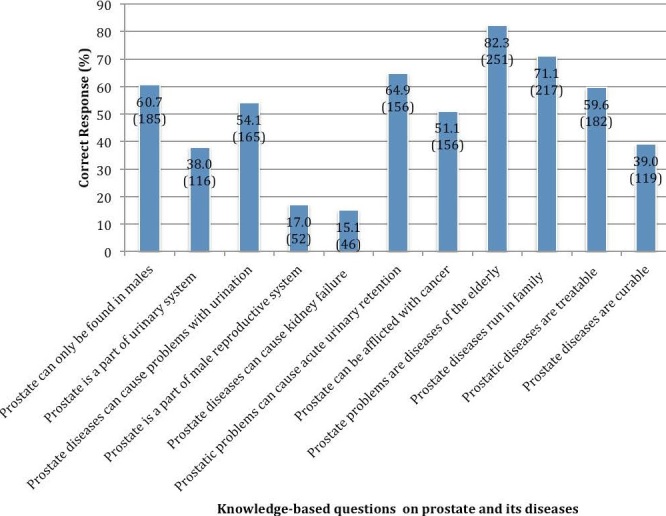
Participants' responses to some knowledge-based questions

***Knowledge of prostatic diseases***: Overall, one hundred and forty-three (46.9%) respondents demonstrated good knowledge about prostate diseases while 162 (53.1%) had poor knowledge. Knowledge of participants on some key questions about prostatic diseases is shown in Figure 1. About 39.0% and 59.7% did not know that prostatic problems are curable and treatable respectively. Also, very few participants knew that prostatic diseases can cause kidney failure (15.1%) and that prostate is part of male reproductive system (17.0%).

***Misconceptions about causes of prostate problems***: Numerous misconceptions and unfounded beliefs about the aetiology of prostatic diseases amongst the participants are depicted in Table 2. Positive history of sexually transmitted diseases was the most common misconception on the aetiology of prostatic diseases in 198 (64.9%) participants.

**Table 2:** Common misconceptions about the aetiology of prostatic diseases

* Misconception	Frequency (n)	Per cent (%)
Positive history of sexually transmitted diseases	196	64.3
Positive history of multiple sexual partners	176	57.7
Excessive sexual activities	116	38.0
Spiritual attack	81	26.6
Married status (married versus single)	76	24.9
Poverty	74	24.3
Polygamous marriage	38	12.5
Early childbearing	40	13.2
Late child bearing	36	11.8

* Multiple responses possible

***Attitudes to screening and treatment of prostatic diseases***: Table 3 shows the responses of respondents to attitude questions regarding prostate diseases screening and treatment. Overall, 42.6% of the respondents had good attitude to screening and treatment of prostatic diseases compared with 57.4% with poor attitude. Details of respondents’ responses are contained in the table.

**Table 3:** Attitudes of the participants to prostatic diseases

Statement	Response					TOTAL n (%)
	Strongly Agree n (%)	Agree n (%)	Indifferent n (%)	Disagree n (%)	Strongly Disagree n (%)	
All adult men should undergo screening for prostate cancer	28 (9.2)	58 (19.0)	61 (20.0)	75 (24.6)	83 (27.2)	305 (100.0)
Early diagnosis of prostate cancer improves the clinical outcome	30 (9.8)	34 (11.2)	68 (22.3)	78 (25.6)	95 (31.1)	305 (100.0)
Early consultation with doctors for urinary symptom is helpful	34 (11.2)	38 (12.5)	60 (19.6)	64 (21.0)	109 (35.7)	305 (100.0)
Drug treatment of prostatic diseases is effective	19 (6.2)	27 (8.9)	30 (9.8)	101 (33.1)	128 (42.0)	305 (100.0)
Medical & surgical treatments can cure prostatic problems	25 (11.5)	30 (23.0)	41 (13.4)	77 (25.2)	132 (26.9)	305 (100.0)
Consultation with doctor is only necessary when home remedy fails	201 (65.9)	47 (15.4)	35 (11.5)	13 (4.3)	09 (3.0)	305 (100.0)

***Prostate cancer screening practices***: Of the 305 respondents, only 57 (18.7%) had ever been advised by a physician to undertake a PSA screening while only 31 (10.2%) respondents had ever carried out PSA screening for PCa. Of the ones who had carried out PSA screening, only 18 (5.9%) had done it more than once while only 9 (3.0%) men usually do PSA on an annual basis. Similarly, only 105 (34.4%) men could remember DRE being performed on them before either for prostate examination or other anorectal complaints. Two hundred and ninety-four (96.4%) respondents were willing to undertake screening for PCa. Reasons given by 274 respondents who had never been screened for PCa were multiple and depicted in Table 4.

**Table 4:** Screening practices and reasons for not undergoing screening amongst participants

Variable/Response	Frequency (n)	Percent (%)
**Had ever been advised to do PSA**		
Yes	57	18.7
No	231	75.7
Don’t know	17	5.6
**Had done a PSA before**		
Yes	31	10.2
No	263	86.2
Don’t know	11	3.6
**Does PSA testing regularly**		
Yes	9	3.0
No	296	97.0
**Has had a DRE done before**		
Yes	105	34.4
No	200	65.6
**Willingness to undertake Screening for CaP**		
Yes	294	96.4
No	11	3.6
*Reasons for not undergoing screening		
They were never advised by physician	192	63.0
They were unaware of screening	156	51.1
Thought no need for screening since no symptoms	126	41.3
Did not know where to go for screening	105	34.4
Thought they could not develop CaP	100	32.8
Lack of interest in screening	22	7.2
Financial constraints	18	5.9
No reason given	24	14.4

* Multiple responses possible

***Influence of socio-demographic data on knowledge, attitudes and screening practices***: Effects of various socio-demographic data on knowledge, attitudes and screening practices were explored. Only educational and occupational status had significant associations with the level of knowledge of as well as attitudes to prostatic diseases. The only factor that influenced screening practices was educational status. Details are depicted in **Table 5**.

**Table 5:** Influence of socio-demographic characteristics on KAP regarding prostatic diseases and screening

Socio-demographic Characteristic	Knowledge		Attitudes		Screening Practices			
	Poor n (%)	Good n (%)	Poor n (%)	Good n (%)	Good n (%)		Poor n (%)	TOTAL N (%)
**Age**								
41-50	31 (53.4)	27 (46.6)	34 (58.6)	24 (41.4)	6(10.3)		52 (89.7)	58 (100.0)
51-60	39 (51.3)	37 (48.7)	41 (53.9)	35 (46.1)	7 (9.2)		69 (90.8)	76 (100.0)
61-70	45 (56.3)	35 (43.7)	48 (60.0)	32 (40.0)	5 (6.3)		75 (93.7)	80 (100.0)
71-80	30 (52.6)	27 (47.4)	32 (56.1)	25 (43.9)	7 (12.3)		50 (87.7)	57 (100.0)
81-90	11 (52.4)	10 (47.6)	12 (57.1)	9 (42.9)	4 (19.0)		17 (81.0)	21 (100.0)
˃ 90	6 (46.2)	7 (53.8)	8 (61.5)	5 (38.5)	2 (15.4)		11(84.6)	13 (100.0)
**P-value**	**0.984**		**0.980**		**0.56**			
**Educational status**								
Nil	52 (81.3)	12 (18.7)	55 (85.9)	9 (14.1)	2 (3.1)		62 (96.9)	64 (100.0)
Primary	45 (64.3)	25 (35.7)	46 (65.7)	24 (34.3)	4 (5.7)		66 (94.3)	70(100.0)
Secondary	44 (44.9)	54 (55.1)	48 (49.0)	50 (51.0)	8 (8.2)		90 (91.8)	98 (100.0)
Tertiary	21 (28.8)	52 (71.2)	26 (35.6)	47 (64.4)	17 (23.3)		56 (76.7)	73 (100.0)
**P-value**	**< 0.001**		**< 0.001**		**< 0.001**			
**Marital status**								
Single	5 (55.6)	4 (44.4)	4 (44.4)	5 (45.6)	1(11.1)	8 (88.9)		9 (100.0)
Married	142 (52.8)	127(47.2	153(56.9)	116 (43.1)	29 (10.8)	240 (89.2)		269 (100.0)
Divorced	6 (54.5)	5 (45.5)	7 (63.6)	4 (36.4)	0(0.0)	11 (100)		11 (100.0)
Widower	9 (56.3)	7 (43.7)	11 (68.8)	5 (31.2)	1 (6.2)	15 (93.8)		16 (100.0)
**P-value**	**0.991**		**0.645**		**0.655**			
**Occupation**								
Farmers	61 (64.2)	34 (35.8)	67 (70.5)	28 (29.5)	9 (8.9)	87 (91.1)		95 (100.0)
Artisans	38 (62.3)	23 (37.7)	46 (75.4)	15 (25.6)	5 (8.2)	56 (91.8)		61 (100.0)
Civil servants	29 (39.2)	45 (60.8)	30 (40.5)	44 (59.5)	7 (9.5)	67 (80.5)		74 (100.0)
Pensioners	18 (48.6)	19 (51.4)	14 (37.8)	23 (62.2)	5 (13.5)	32 (86.5)		37 (100.0)
Business men	8 (32.0)	17 (68.0)	9 (36.0)	16 (64.0)	4 (16.0)	21 (84.0)		25 (100.0)
Others	8 (61.5)	5 (38.5)	9 (69.2)	4 (30.8)	1 (7.7)	12 (92.3)		13 (100.0)
**P-value**	**< 0.010**		**<0.001**		**0.87**			
**Religion**								
Christianity	134(52.1)	123(47.9)	143 (55.6)	114(44.4)	28 (10.9)	229(75.1)		257 (100.0)
Islam	7(56.7)	13 (43.3)	19 (63.3)	11 (36.7)	3 (10.0)	27 (90.0)		30 (100.0)
Traditional religion	11 (61.1)	7 (38.9)	13 (72.2)	5 (27.3%)	0 (0.00)	18 (100.0)		18 (100.0)
**P-value**	**0.700**		**0.305**		**0.34**			

***Correlation between knowledge and attitude of participants***: Using Pearson's correlation, knowledge and attitudes towards prostatic diseases were positively correlated with each other as 73.4% participants with good knowledge had positive attitude and 84.6% with poor knowledge had negative attitude to prostatic diseases (r = 0.585, p < 0.001).

## Discussion

The most common diseases of the prostate namely BPH and PCa are among the most common afflictions of the elderly and constitute a major reason for urologic referral in this age group. They are major causes of lower urinary tract symptoms (LUTS) with its attendant complications and adverse effects on quality of life. Two notable technological advances have greatly contributed to the startling increase in the awareness and diagnosis of prostate diseases. These are availability of serum-based assays, which have been developed for the measurement of prostate specific antigen (PSA) and widespread availability and affordability of ultrasound scanning which usually provide an excellent imaging of a formerly poorly visualized prostate gland [12]. Of these two advances, however, measurement of PSA has had the most significant effects. Over the past decade, these assays have completely revolutionized the approach to PCa-from initial screening and staging through to monitoring during therapy [13]. The results of this study showed an average level of awareness of prostatic diseases but overall level of knowledge was poor as only 47% of the participants had good knowledge about prostatic diseases. Though the level of awareness was still poor, it however represents an improvement on the earlier reports from Nigeria [11, 14]. About a quarter of the participants in our study had heard about PSA screening compared to 5.8% documented in an earlier study [11]. This finding is very similar to the awareness level reported in an Ugandan population-based study some years earlier [15]. In addition, about two-thirds have heard of PCa, which is also an improvement on the earlier findings in Nigeria. This is probably due to increasing use of PSA for screening and increasing public health enlightenment about PCa in recent times. However, information about benign prostatic conditions namely BPH and prostatitis was still very poor.

A lot more has to be done to educate people about these diseases, as 48.2% of participants in this study had never heard about any of the three main prostatic diseases. Surprisingly, 67.5% had not heard about BPH, which is the most common prostatic disease. They claimed to have heard about it for the first time during the publicity for data collection for this project. Despite the growing trend of increased uptake of screening for PCa worldwide, less than one third of the respondents in our study knew about screening for PCa while only a quarter had heard about PSA screening [16, 17]. Amongst the respondents who had previous information about prostate and prostatic diseases, the most common sources of information were radio, television and newspapers. It is surprising that fewer respondents got their information from the hospitals that are supposed to be a major source of information for diseases like this. This might be due to emphasis on curative treatment rather than preventive which makes general practitioners (GPs) in Nigeria concentrate on the presenting complaints rather than holistic assessment of the patients and recommendation of other tests relevant to the patient's age and clinical demands [18]. Enlightening patients on other medical conditions they are at risk of developing during consultation for another illness will help in early detection of other diseases and improve prognosis particularly for malignancies. The religious institutions did not contribute much to dissemination of information to patients about prostatic diseases, as religious worship centres were sources of information in less than 15% of participants. The findings of this study also suggest that the mass media have also not fared well as expected in dissemination of information about prostatic problems to the populace. The best of these mass media was radio, which was the source of information for 59.5% of those who had heard about prostatic diseases before which translates to only 30.8% of the entire participants. Therefore, a widespread public health education using the mass media, hospitals and religious centres is advocated to encourage early presentation of men suffering from prostatic diseases, which ultimately will reduce the morbidity and mortality rates of these diseases.

About three-quarter of the participants were aware of the positive role of age and family in the development of prostate pathologies. However, knowledge about prostate being part of male reproductive system and prostate diseases as causes of renal failure was abysmally low. In this study, there were many erroneous misconceptions about the aetiology of prostatic diseases. Positive history of sexually transmitted diseases, multiple sexual partners and excessive sexual activities stood out as the three most common misconceptions. Though there are no scientific bases for these beliefs, more than half of the study participants hold these erroneous beliefs. These beliefs are not peculiar to Nigerian men as respondents in a study in Burkina Faso gave similar reasons [19]. Furthermore, as many as a quarter of the respondents believed that poverty and spiritual attacks can cause prostatic diseases. It is also shocking that about 61% believed that prostatic diseases are incurable. It is evident from this study that a lot of education is required to disabuse the minds of men in this environment from these erroneous and incorrect beliefs, as this may have a negative impact on their healthcare-seeking attitudes and behaviour. Emphasis should therefore be placed on health educational intervention programs that will be geared towards improving men's awareness and knowledge. In our study, 42.6% of respondents had good attitude to treatment of prostatic diseases and screening while only 10.2% had ever carried out PSA screening for PCa. Only 3.0% of the men who had done PSA before usually do PSA testing on an annual basis. Though these figures represent an improved uptake of PSA testing compared to earlier studies in our environment, however it is still a far cry from the expected after availability of PSA as a screening tool for about four decades [11, 14]. Similarly, only about 34.4% of the men could remember DRE being performed on them before. This shows the underutilization of DRE as a screening tool for PCa by the GPs, which may lead to non-detection or failure to detect PCa cases at an early stage [20]. In this study, educational status was found to be associated with knowledge and attitudes towards prostatic diseases as respondents with higher level of education were more likely to have positive attitude to treatment of prostate diseases and screening. Furthermore, occupation also played a positive role as civil servants, business men and retirees were found to have better level of awareness as well as more positive attitude to prostatic diseases than people of other occupations. Notably, these occupations are also associated with higher levels of education. This is in keeping with the findings amongst African Americans where socio-economic status was found to significantly affect the level of knowledge and attitudes with people of higher socio-economic factors having a higher level of awareness and knowledge about PCa and more positive attitude and behaviour towards screening for CaP [21].

This study also found a positive correlation between the levels of knowledge and attitudes to prostatic diseases. Most of the participants with good knowledge correspondingly had positive attitude and vice versa. This also concurs with previous reports [9, 21, 22]. In this study, the most common reason why most of the men have never done any screening for PCa was lack of recommendation from primary care physicians. More than three-quarter of respondents had never been asked to do PCa screening by their primary care physicians and more than half gave this as a reason not to have carried out a PSA testing. This underscores the role of GPs in improving the uptake of PCa screening. Interestingly, almost all (94.6%) of the men in this study were willing to undertake screening for PCa if recommended. Therefore, it is important for GPs to show interest and recommend screening for men at risk when encountered in their practices. Most of the other reasons given by respondents for not embracing screening for prostate cancers were centred on poor knowledge about prostatic diseases which again emphasizes the importance of efforts directed at improving awareness and knowledge. Finally, this study also shows that poverty contributes to poor attitude and screening practices amongst participants. This is in keeping with other studies, which demonstrated a strong link between poverty and mortality from non-communicable diseases [23, 24]. Mortality from these diseases might not be unconnected with possible late presentations stemming from financial constraints and inability to afford optimal care. It is therefore almost certain that measures to improve poverty will indirectly enhance positive attitude and healthcare-seeking behaviour.

## Conclusion

In conclusion, the results of this present study suggest that the level of awareness about prostatic diseases remains low among the men population in Nigeria with main sources of information being radio and television programmes. Not many respondents got their information from the health personnel contrary to expectation. This study also established a high level of erroneous beliefs about the aetiology of prostatic diseases with positive history of sexually transmitted diseases being the most common misconception regarding the aetiology of prostatic diseases. The level of knowledge and attitudes regarding screening and treatment of prostatic diseases were both low and were influenced by level of education and occupational status. Screening practices were found to be abysmally poor and were influenced by level of education of respondents only. This study recommends widespread public health campaigns using the mass media, hospitals and religious centres to improve knowledge, attitude and screening practices regarding prostatic diseases.

### What is known about this topic

- It has been well established that prostate cancer is the most common cancer in men beyond middle age and that prevalence and mortality of this disease are higher in black men;
- Screening for prostate cancer in at risk men ensures early diagnosis and treatment. However, studies have shown that majority of patients present with advanced diseases in Nigeria due to lack of organized screening;
- The level of awareness of prostate cancer and screening practices are generally low in Nigeria and this may partly be responsible for late presentation.

### What this study adds

- This study adds to the body of evidence that awareness of prostate cancer and screening practices are still poor in Nigeria. It also establishes that level awareness of other prostatic diseases namely benign prostatic hyperplasia and prostatitis is even lower than that of prostate cancer;
- It was also discovered that information about prostatic diseases are not commonly obtained from the medical personnel. Furthermore, the failure of medical personnel to recommend screening for prostate cancer is the most common reason why men have not been screened. This study recommends that doctors should utilize holistic approach to patient's management and recommend relevant screening in patients at risk;
- This study also establishes some misconceptions about aetiology of prostatic diseases and need for public enlightenment to disabuse the mind of men on these erroneous beliefs to enhance positive healthcare-seeking attitudes and behaviours.

## Competing interests

The authors declare no competing interest.
